# Defining and Measuring Diagnostic Uncertainty in Medicine: A Systematic Review

**DOI:** 10.1007/s11606-017-4164-1

**Published:** 2017-09-21

**Authors:** Viraj Bhise, Suja S. Rajan, Dean F. Sittig, Robert O. Morgan, Pooja Chaudhary, Hardeep Singh

**Affiliations:** 1 Center for Innovations in Quality, Effectiveness and Safety, Michael E. DeBakey Veterans Affairs Medical Center and Baylor College of Medicine, Houston, TX USA; 2 School of Public Health, University of Texas Health Science Center, Houston, TX USA; 3 School of Biomedical Informatics, University of Texas Health Science Center, Houston, TX USA; 4 UT-Memorial Hermann Center for Health Care Quality and Safety, Houston, TX USA

**Keywords:** diagnostic uncertainty, diagnostic process, measurement, definition, review

## Abstract

**Background:**

Physicians routinely encounter diagnostic uncertainty in practice. Despite its impact on health care utilization, costs and error, measurement of diagnostic uncertainty is poorly understood. We conducted a systematic review to describe how diagnostic uncertainty is defined and measured in medical practice.

**Methods:**

We searched OVID Medline and PsycINFO databases from inception until May 2017 using a combination of keywords and Medical Subject Headings (MeSH). Additional search strategies included manual review of references identified in the primary search, use of a topic-specific database (AHRQ-PSNet) and expert input. We specifically focused on articles that (1) defined diagnostic uncertainty; (2) conceptualized diagnostic uncertainty in terms of its sources, complexity of its attributes or strategies for managing it; or (3) attempted to measure diagnostic uncertainty.

**Key Results:**

We identified 123 articles for full review, none of which defined diagnostic uncertainty. Three attributes of diagnostic uncertainty were relevant for measurement: (1) it is a subjective perception experienced by the clinician; (2) it has the potential to impact diagnostic evaluation—for example, when inappropriately managed, it can lead to diagnostic delays; and (3) it is dynamic in nature, changing with time. Current methods for measuring diagnostic uncertainty in medical practice include: (1) asking clinicians about their perception of uncertainty (surveys and qualitative interviews), (2) evaluating the patient–clinician encounter (such as by reviews of medical records, transcripts of patient–clinician communication and observation), and (3) experimental techniques (patient vignette studies).

**Conclusions:**

The term “diagnostic uncertainty” lacks a clear definition, and there is no comprehensive framework for its measurement in medical practice. Based on review findings, we propose that diagnostic uncertainty be defined as a “subjective perception of an inability to provide an accurate explanation of the patient’s health problem.” Methodological advancements in measuring diagnostic uncertainty can improve our understanding of diagnostic decision-making and inform interventions to reduce diagnostic errors and overuse of health care resources.

**Electronic supplementary material:**

The online version of this article (10.1007/s11606-017-4164-1) contains supplementary material, which is available to authorized users.

## INTRODUCTION

Diagnostic uncertainty is inherent in the practice of medicine. Patients often present with undifferentiated symptoms that change over time, making it difficult for clinicians to identify a satisfactory explanation of the patient’s presenting problem.^1^–^5^ In addition to patient presentation, time constraints of the patient–clinician encounter, complexity of medical science and limitations of diagnostic tests all influence diagnostic decisions in the midst of uncertainty.^6^–^8^ Described as analogous to looking for a “snowball in a blizzard,” diagnostic decision-making under uncertainty is challenging for clinicians and must be appropriately managed in medical practice.^1^

In previous studies, diagnostic uncertainty has been associated with diagnostic variation (physicians giving different diagnoses to the same patient), over-testing, unnecessary surgeries, more hospitalizations and referrals, and increased health care expenditure.^9^–^15^ Inappropriate management of diagnostic uncertainty could contribute to diagnostic errors or excess health care utilization.^3^,^16^–^19^ Recent estimates suggest that at least 1 in 20 outpatients experience a diagnostic error (missed, delayed or incorrect diagnoses) each year, sometimes with devastating consequences.^20^,^21^ Additionally, rising costs related to diagnostic testing have led to recommendations for cost-containment, requiring physicians to carefully consider the resources they use and diagnostic decisions they make in the midst of uncertainty.^12^,^22^–^25^ Thus, inappropriate management of diagnostic uncertainty can impact both system and patient outcomes.^11^,^16^,^17^,^26^

The “basic science” of diagnostic uncertainty is poorly understood.^2^,^5^,^27^–^29^ To our knowledge, diagnostic uncertainty has yet to be adequately conceptualized in medical practice, and few efforts have been made to measure it in clinical settings.^2^,^5^,^27^–^29^ This knowledge is foundational for the development of interventions to identify and manage it appropriately. To this end, we synthesized existing literature to ascertain how diagnostic uncertainty has been defined in medical practice, and what assessment methods are used for its measurement.

## METHODS

### Data Sources and Search Strategy

We used multiple search strategies to identify candidate articles describing uncertainty pertaining to diagnosis in medical practice. We conducted a systematic search of the OVID Medline and PsycINFO databases using a combination of keyword searches and medical subject headings (MeSH); see full list in supplemental files. Our search included all publications through May 29 2017, with no restrictions on publication type (journal articles, books, etc.) or geography. We limited our search to English-language publications that focused on humans and had abstracts that could be used for initial screening. Our primary search yielded 7024 articles.

To ensure that we did not miss any published literature, we used multiple secondary search strategies to locate additional relevant articles for review. First, we manually reviewed the references of the articles identified in the primary search. Second, we searched a topic-specific database (Agency for Health Care Research and Quality’s PSNet) with a subset of terms listed in the supplemental file. Third, we identified additional references by contacting authors and several experts in the field of diagnostic error, diagnostic uncertainty and clinical reasoning. Together, these secondary search methods yielded an additional 131 articles.

### Selection Strategy, Data Extraction and Categorization

**Figure 1:**
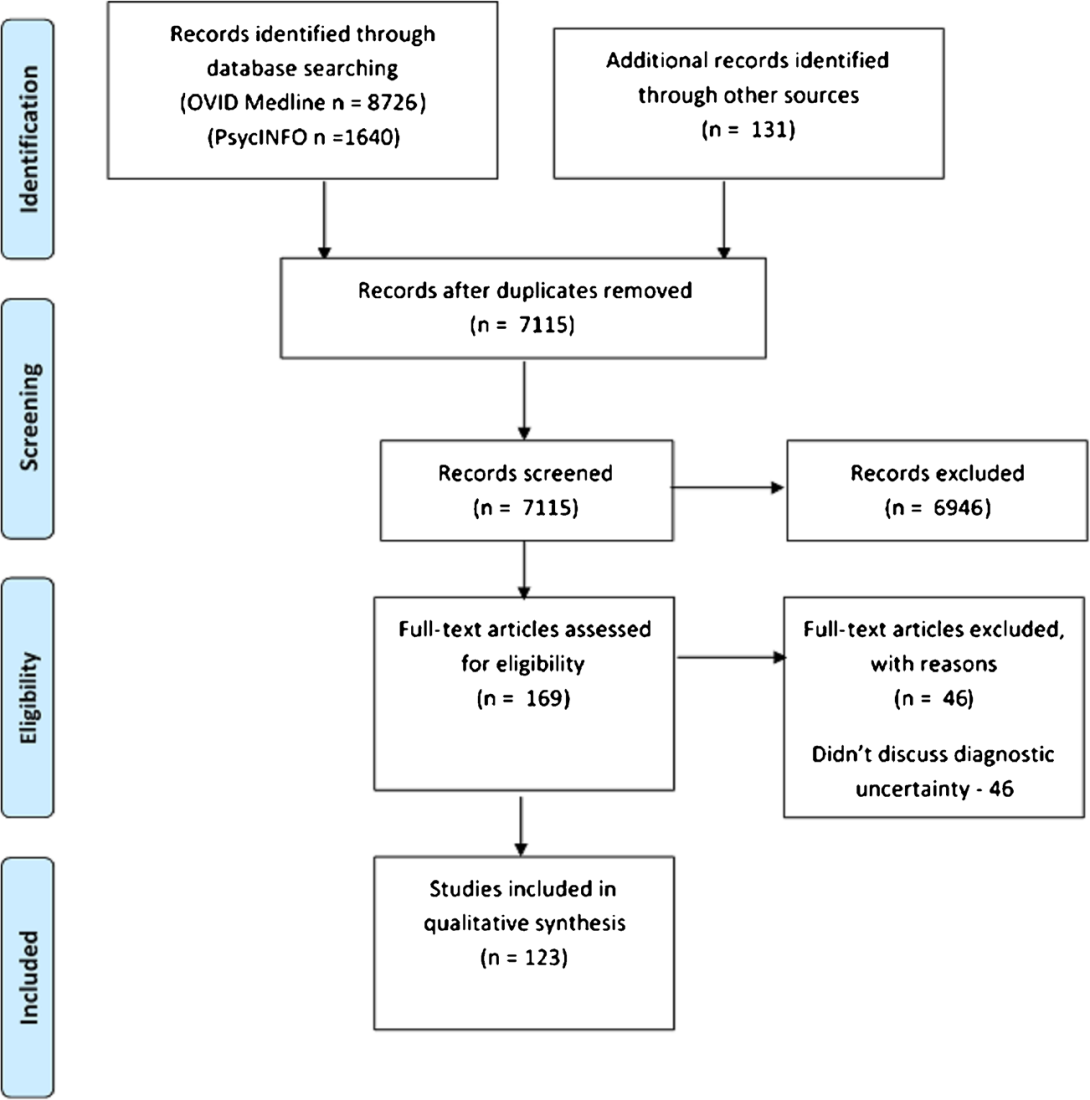
Flow diagram highlighting the database search, screening and inclusion.

Because diagnostic uncertainty has not been well studied, we included all types of articles—original research articles, reviews, editorials, perspectives, commentaries and case reports—that described uncertainty in the diagnostic process. Only articles discussing diagnosis-related uncertainty in medical practice, i.e., uncertainty experienced by physicians, nurses, registered nurse practitioners or physician assistants, were included. We specifically focused on articles that (1) defined diagnostic uncertainty; (2) discussed diagnostic uncertainty in terms of its sources, complexity of its attributes or strategies for managing it; or (3) attempted to measure diagnostic uncertainty. We excluded studies that did not discuss uncertainty specifically in the process of diagnosis (Fig. 1, PRISMA Flowchart).

Abstracts were independently reviewed by two physicians (VB and PC) with expertise in health services research and were marked as included or excluded. Disagreements were resolved by team consensus. Reviewers then independently examined all included full-text articles qualitatively and categorized them according to the primary information they provided: (1) definition, (2) conceptual understanding of diagnostic uncertainty (sources, attributes or management strategies for diagnostic uncertainty), (3) measurement method or (4) other. To improve reliability and consistency, reviewers first independently reviewed 20 abstracts as a pilot, compared categorization, and then refined categorization criteria. All included articles were categorized by both reviewers, and disagreements in categorization were resolved by team consensus. We also categorized articles according to measurement method: (1) studying clinicians’ subjective perceptions of uncertainty, (2) evaluating the patient–clinician encounter or (3) using experimental techniques. We piloted the use of the validated Downs and Black checklist^30^ to assess study quality and bias, but because very few studies were interventional, and approaches among studies were highly heterogeneous, we did not pursue this assessment.

## RESULTS

We identified 123 articles on diagnostic uncertainty that met the criteria for full review, and these are discussed below in detail.

### Defining Diagnostic Uncertainty

Although none of the articles defined *diagnostic uncertainty*, multiple experts in medicine, psychology, nursing, anthropology and sociology alluded to uncertainty in the context of diagnosis while defining uncertainty more generally in clinical practice.^31^–^33^ In these studies, diagnostic uncertainty was discussed as a perception of not knowing something (lack of knowledge); Politi et al. and Mishel et al. described *uncertainty* as the inability to determine the meaning of illness-related events.^34^,^35^ Han, Greenhalgh and Sommers defined *uncertainty* as a subjective perception of ignorance or not knowing.^31^,^33^,^36^ Other definitions are described in Table 1.

**Table 1:** Definitions of Uncertainty in Medicine with Relevance to Diagnostic Decision-Making

Authors, Publication Year	Article Type	Brief Synopsis of Key Concepts Discussed	Definition
Mishel, 1988^34^	Literature review	Definition of uncertainty and taxonomy of uncertainty	The inability to determine the meaning of illness-related events
Penrod, 2000^37^	Literature review	Conceptual attributes of uncertainty, evolution of the concept of uncertainty and issues surrounding measurement	Uncertainty is a dynamic state in which there is a perception of being unable to assign probabilities for outcomes that prompts a discomforting, uneasy sensation that may be affected through cognitive, emotive or behavioral reactions or by passage of time and changes in the perceptions of circumstances. The experience of uncertainty is pervasive in human existence and is mediated by feelings of confidence and control that may be highly specific (event-focused) or more global (a worldview).
Politi, 2007^35^	Literature review	Conceptualizing uncertainty, sources of uncertainty and outcomes of communicating uncertainty	The inability to determine the meaning of illness-related events resulting from ambiguity, complexity, unpredictability of illness, deficiency of information about one’s illness and its consequences
Han, 2011^33^	Literature review	Issues and sources of uncertainty; taxonomy of uncertainty, and definition of uncertainty	The subjective perception of ignorance
Cousin, 2013^38^	Experimental study	Operational definition of uncertainty for creation of vignettes with uncertainty	State of not knowing something accurately or precisely, or as a lack of confidence in one’s knowledge of something
Seely, 2013^39^	Literature review	Manifestations and implications of uncertainty	Relative degree of our ability to predict the future. Viewed as a dynamic and variable function of time, capable of stable or erratic variation
Greenhalgh, 2013^31^	Book chapter	Different perspectives on uncertainty in clinical practice	A subjective perception of not knowing what to think or what to do
Sommers, 2013^31^	Book chapter	Introduction to uncertainty in primary care, three theoretical perspectives addressing the concept of uncertainty, and an operational definition of uncertainty	The confusion, conflict, stuckness, unease and/or discomfort an individual primary care clinician experiences when confronting a predicament in an individual patient who presents a diagnostic dilemma

### Conceptualizing Diagnostic Uncertainty

Three salient attributes of diagnostic uncertainty were discussed in the literature. First, diagnostic uncertainty is a perception or an emotional response, highlighting the subjective component of experiencing it in medical practice.^31^–^33^,^40^,^41^ Clinicians can, therefore, experience varying degrees of diagnostic uncertainty, depending on their training, past experiences and risk tolerance.^12^,^41^–^44^ Second, diagnostic uncertainty impedes the clinician’s ability to act or think appropriately to initiate definitive treatment for the stated problem.^31^,^36^ This reflects the complexity of medical science, as well as the process of diagnosis, which involves narrowing a broad list of potential diagnoses into fewer options as more information is gathered, interpreted and integrated.^21^,^31^,^36^ Clinicians are unable to initiate definitive treatment while trying to reduce their diagnostic uncertainty through various options—conducting a more detailed patient interview, ordering more diagnostic tests, initiating referrals, scheduling close follow-up appointments, choosing a risk-averse disposition (in-hospital admission or referral to the emergency room), testing a particular treatment strategy or even deferring the decision (test of time).^2^,^13^,^14^,^37^,^42^,^45^–^49^ Mismanagement of diagnostic uncertainty can thus potentially contribute to both diagnostic delays and over-testing/or treatment.^3^,^16^,^17^ Third, diagnostic uncertainty is dynamic and changes with time.^37^,^39^ For a patient presenting early in the course of disease (with undifferentiated symptoms), a clinician might have considerable uncertainty that may resolve with time as details evolve.^3^ Additionally, patient interactions with other components of the health system (clinician and specialist visits, diagnostic testing and therapy) might provide relevant diagnosis-related information, which can influence diagnostic uncertainty (increase or decrease it).^50^,^51^

Multiple taxonomies of uncertainty in medical practice have been discussed in the literature.^32^,^52^ For example, Fox categorized uncertainty derived from personal academic limitations and from the limits of existing knowledge.^53^,^54^ Beresford categorized it as technical uncertainty (paucity of adequate information or scientific data to predict the effects of certain factors in the progress of a disease), conceptual uncertainty (challenges in applying population-based knowledge in relation to a particular patient) and personal uncertainty (role of interpersonal relationships between the patient, provider and other medical personnel involved in care influencing the diagnostic process and causing uncertainty).^31^–^33^,^55^–^58^ Han described uncertainty using three principles—probability, ambiguity and complexity—and provided a taxonomy to measure sources and issues related to uncertainty.^33^,^59^,^60^ Greenhalgh described four dimensions of uncertainty relevant to clinical practice: 1) uncertainty in the “voice of medicine” relating to the completeness, accuracy and relevance of research-based evidence in medical science; 2) uncertainty in the patient’s story; 3) uncertainty about what best to do for a particular patient; 4) and uncertainty arising from complex collaborative endeavors in clinical care.^28^ These taxonomies and conceptual approaches highlight key concepts and sources of diagnostic uncertainty in medical practice.

### Methods for Measuring Diagnostic Uncertainty

We found 39 studies describing different methods of measuring diagnostic uncertainty in clinical practice (see Tables 2 and 3), but no comprehensive measurement framework. Previous methods include asking clinicians about their perceptions of uncertainty (surveys and qualitative interviews), evaluating the patient–clinician encounter (such as by reviews of medical records, transcripts of patient–clinician communication and observation) and experimental techniques (patient vignette studies).

**Table 2:**
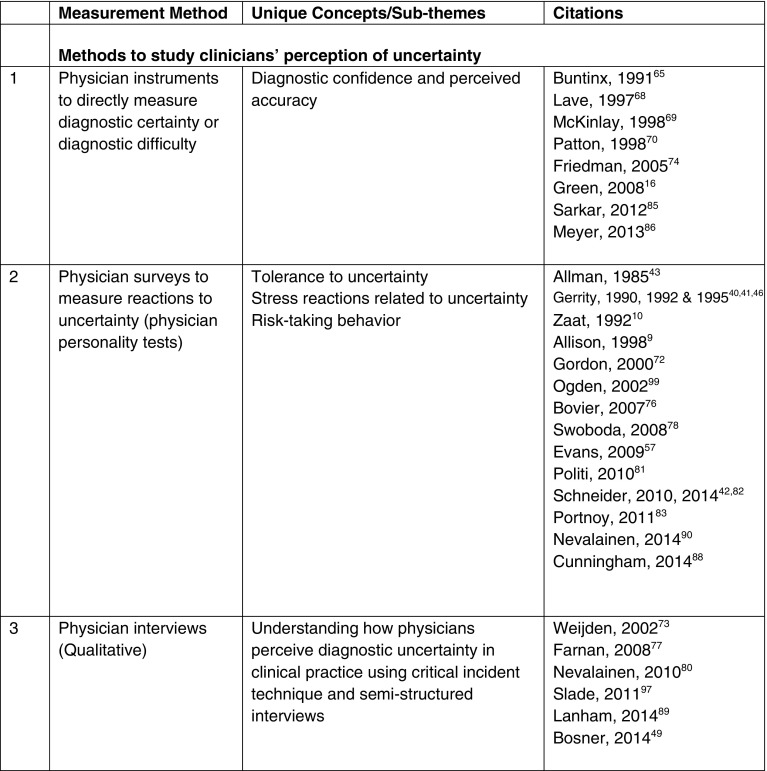

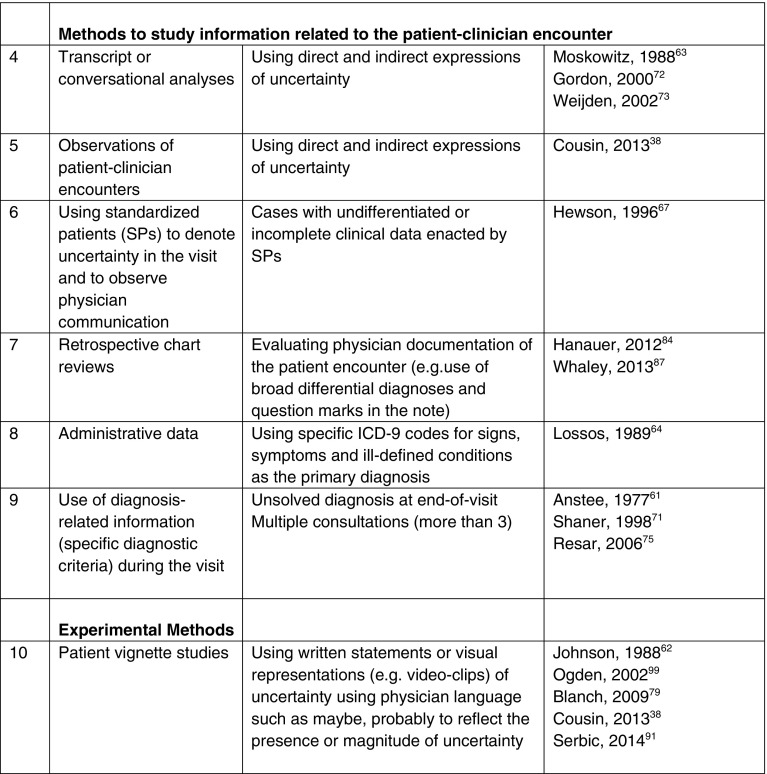
Methods Used to Measure Diagnostic Uncertainty in Clinical Practice

**Table 3:** Methodological Details of Studies Measuring Diagnostic Uncertainty

Author, Publication Year	Article Type and Study Design	Study Objectives	Measurement Methods and Tools Used
Anstee, 1977^61^	Longitudinal hospital-based study	Objective: To follow up patients with diagnostic uncertainty and to understand clinical and demographic factors associated with it	Unsolved diagnosis at discharge from general inpatient unit
Allman, 1985^43^	Physician survey	Objective: To gain insight into the individual physician’s tolerance for diagnostic uncertainty	Pre- and post-liver-spleen scan probability estimates provided by physicians were used for identification of diagnostic uncertainty.
Johnson, 1988^62^	Vignette study with patients	Objective: To evaluate the impact of communicating uncertainty on patient visit satisfaction	Video clips of patient–physician encounters denoting physician expressions of uncertainty were shown to patients, and a questionnaire was subsequently used to evaluate patient responses.
Moskowitz, 1988^63^	Transcript of the physician’s encounter	Objective: To generate hypotheses regarding how physicians make difficult clinical decisions	Case presentations with details about difficult diagnoses were presented to 3 physicians and were used as measures for the presence of uncertainty.
Lossos, 1989^64^	Retrospective analysis of patient records and administrative data	Objective: To operationalize a definition for deferred diagnosis (when diagnosis is unclear) and describe its clinical spectrum	The primary diagnosis for the visit denoted with ICD-9 codes for signs, symptoms and ill-defined conditions was used to identify uncertainty.
Buntinx, 1991^65^	Physician survey	Objective: To compare the initial diagnosis made immediately after physical examination by a general practitioner with final diagnosis made between 2 weeks and 2 months later	Initial diagnosis was captured with a score on the certainty of the diagnosis: “unknown,” “suspected,” “probable” or “certain.”
Gerrity, 1990, 1992 & 1995^40^,^41^,^66^	Physician survey	Objective: Development and refinement of an instrument to measure uncertainty in physicians	23-item Physicians’ Reaction to Uncertainty (PRU) scale was developed, containing subscales for stress from uncertainty, anxiety and reluctance to disclose uncertainty.
Zaat, 1992^10^	Physician survey	Objective: To explore the relationship between physician uncertainty, risk-taking attitude and laboratory test use	Three categories reflecting different levels of diagnostic uncertainty (uncertain, moderately certain and certain) were evaluated using a questionnaire with two 5-point scales. The first scale (14 items) evaluated the extent of the physician’s self-reproach. The second scale (5 items) assessed opinions about risk avoidance.
Hewson, 1996^67^	Study of patient–physician encounter using standardized patients	Objective: To identify strategies involved in diagnosis and treatment plans for primary care problems that are uncertain and complex	Study of primary care physician interactions with standardized patients who portrayed typical primary care problems involving uncertainty and complexity
Lave, 1997^68^	Physician survey	Objective: To determine whether hospital staff’s diagnostic uncertainty is a predictor of hospital resource use	A visual analog scale (0, 25, 50, 75 and 100) was used to measure diagnostic uncertainty.
Allison, 1998^9^	Physician survey	Objective: To explore the association between PCPs’ attitudes toward risk-taking and uncertainty	A 23-item Physicians’ Reaction to Uncertainty (PRU) scale was used.
McKinlay, 1998^69^	Physician survey	Objective: To determine the presence of diagnostic uncertainty and its association with variation in diagnosis and patient factors	A scale of uncertainty from 0 to 100 was used.
Patton, 1998^70^	Physician survey	Objective: To develop a 5-point scale to identify uncertainty in pediatrics	Levels of certainty of diagnosis were captured: very little, some, moderate, substantial, sufficient.
Shaner, 1998^71^	Retrospective analyses of patient charts	Objective: to determine the sources and frequency of diagnostic uncertainty for patients with chronic psychosis and active cocaine abuse or dependence	Cases where a provisional diagnosis or a list of alternate diagnoses was used
Gordon, 2000^72^	Physician survey Transcript analyses using audiotapes of patient–physician encounters	Objective: To identify physician expressions of uncertainty during actual clinic visits and to examine their associations with physicians’ and patients’ characteristics and communication behaviors	A 23-item Physicians’ Reaction to Uncertainty (PRU) scale was used. Physician expression of uncertainty was determined by trained coders. Direct and unambiguous statements included “I do not know” or “it’s not clear.” Indirect expressions (e.g., it was “pretty much” normal or there is a “good chance” it’s normal) were not used due to lack of reliability.
Ogden, 2002^99^	Physician perspectives about behavioral and verbal expressions of uncertainty Vignette study with patients	Objective: To explore the impact of doctors’ expressions of uncertainty for a range of diagnostic and management aspects of the consultation	Behavioral expressions of uncertainty were used—e.g., used a book or computer to find out about a condition or a drug, asked another GP or nurse for advice or referred the patient to a hospital. Verbal expressions of uncertainty were also used—e.g., “I am not sure about this,” I need to find out more,” “let’s see what happens,” “I do not know,” “I have not come across this before,” “I think this might be….” Patients rated the expression of uncertainty.
Weijden, 2002^73^	Qualitative interview with physicians regarding the patient–provider encounter	Objective: To understand the general practitioner’s (GP’s) motives for ordering laboratory tests for patients presenting with unexplained symptoms	Qualitative assessment of physicians’ perceptions of specific diagnosis was conducted immediately after a consultation with a patient.
Friedman, 2005^74^	Physician survey	Objective: To explore the alignment between physicians’ confidence in their diagnoses and the “correctness” of these diagnoses, as a function of clinical experience, and whether subjects were prone to over- or under-confidence	To assess confidence, physicians rated the likelihood that they would, at the time they generated the differential, seek assistance in reaching a diagnosis.
Resar, 2006^75^	Retrospective analyses of patient charts	Objective: Use of a trigger tool to identify adverse events in ICUs	Use of more than 3 consultations as an indicator of diagnostic uncertainty
Bovier, 2007^76^	Physician survey	Objective: To describe sociodemographic and professional characteristics of reactions to uncertainty among physicians from all specialties, including physicians in training	Reaction to medical care uncertainty was measured with the Anxiety Due to Uncertainty and Concern About Bad Outcomes scales.
Green, 2008^16^	Physician survey	Objective: To explore the association between the presence of diagnostic uncertainty and adverse events	A certainty estimate of either ≤20% or ≥80% was classified as clinical certainty, while estimates between 21% and 79% were defined as clinical uncertainty.
Farnan, 2008^77^	Qualitative interview of physicians	Objective: To describe types of uncertainty faced by residents and strategies employed to manage uncertainty and effects on patient care	Using critical incident technique, residents were asked to recall important clinical decisions during a recent call night, with probes to identify decisions made during uncertainty.
Swoboda, 2008^78^	Physician survey	Objective: To examine the clinical decision-making involved in diagnosing contested illnesses (chronic fatigue syndrome, multiple chemical sensitivities and Gulf War syndrome)	Physician respondents were asked whether sufficient knowledge existed for determining legitimacy of contested illnesses.
Blanch, 2009^79^	Patient vignette study	Objective: To examine the consequences of expressions of uncertainty (EOUs) in medical student interactions, with a particular focus on the gender of the expressor	Videotaped interactions were shown to patients. Expressions of uncertainty were used to denote uncertainty. The patients were asked to rate whether the doctor sounded sure of himself/herself on a 10-point scale from 1 = definitely no to 10 = definitely yes.
Evans, 2009^57^	Physician survey	Objective: To investigate the relationship between primary care physician’s stress reactions to uncertainty and conceptual resource of epistemology	Stress reactions to uncertainty were measured using subscales of the Physician Reaction to Uncertainty Scale (PRUS): “Anxiety due to uncertainty” (5 items) and “Concern about bad outcomes” (3 items).
Nevalainen, 2010^80^	Qualitative study with medical students	Objective: To investigate how medical students experience uncertainty during their first clinical years and how their feelings develop with time as they progress from the 3rd to the 4th year	Qualitative assessment of uncertainty in reflective learning diaries and writings collected during 3rd and 4th years of medical studies
Politi, 2010^81^	Physician survey	Objective: To identify variables associated with physicians’ anxiety from uncertainty and reluctance to disclose uncertainty to patients	Scales for “anxiety from uncertainty” and “reluctance to disclose uncertainty” were used.
Schneider, 2010, 2014^42^,^82^	Physician survey	Objective: Development and refinement of the Dealing with Uncertainty Questionnaire (DUQ) and the Communicating and Dealing with Uncertainty (CoDU) questionnaire	The Dealing with Uncertainty Questionnaire (DUQ) was refined, and 4 CoDU scales were identified: “communicating uncertainty,” “diagnostic action,” “intuition” and “extended social anamnesis.”
Portnoy, 2011^83^	Physician survey	Objective: To explore the association between physicians’ attitudes about communicating and managing scientific uncertainty, and their perceptions of negative patient reaction to uncertainty	Four items focusing on physicians’ attitudes toward communicating and managing scientific uncertainty on a 5-point Likert scale from “strongly agree” to “strongly disagree.” Ambiguity Aversion in Medicine Scale was also used.
Hanauer, 2012^84^	Retrospective analysis of patient EHR notes	Objective: Quantified the use of uncertainty expressions from institutions’ EHR	Diagnostic uncertainty identified using 313 distinct uncertainty expressions, e.g., “could be,” “most likely,” “probably,” in the EHR note
Sarkar, 2012^85^	Clinician survey	Objective: To survey primary care practitioners about potential barriers to timely diagnosis in outpatient settings and diagnostic difficulty faced in their practices	Extent of perceived diagnostic difficulty (uncertainty) was determined using the question, “In the past year, about what percentage of your patients did you consider difficult to diagnose?”, with 5 ordered responses of 0%, 1–5%, 6–10%, 11–15% and >15%.
Cousin, 2013^38^	Experimental study with patients Field study with videos of physician during patient–physician encounter	Objective: To determine how physician-expressed uncertainty affects patient satisfaction in relation to both the physician and patient gender	Vignettes using statements of physician expression of uncertainty (e.g., “I cannot tell you” or probability words such as “maybe,” “probably”) Independent coder rated the presence of diagnostic uncertainty using 11-point Likert scale (0 = no uncertainty, 10 = total uncertainty).
Meyer, 2013^86^	Physician vignette study	Objective: To evaluate how physicians’ diagnostic calibrations, defined as the relationship between diagnostic accuracy and confidence in that accuracy, change with evolution of the diagnostic process and with increasing diagnostic difficulty of clinical case vignettes	Diagnostic confidence was measured on a scale of 0 to 10.
Whaley, 2013^87^	Retrospective analysis of patient EHR notes	Objective: To measure antibiotic prescribing rate, prevalence of diagnostic complexity and uncertainty that clinicians face when treating patients with acute cough	Documentation in the EHR note was used to evaluate the presence of diagnostic uncertainty, e.g., use of words such as “maybe” or “unclear” or question marks in association with the diagnosis. Differential diagnosis was also used (e.g., pneumonia vs. acute bronchitis).
Bosner, 2014^49^	Qualitative interview of physicians	Objective: To study the early diagnostic phase of the decision-making process when no specific diagnosis is reached in patients presenting with headache	Qualitative assessment of physicians’ perceptions of uncertainty
Cunningham, 2014^88^	Physician survey	Objective: To investigate whether physician anxiety due to uncertainty is associated with a higher propensity to use race in medical decision-making	Anxiety Due to Uncertainty (ADU), a 5-item measure of emotional reactions to clinical uncertainty, was used.
Lanham, 2014^89^	Qualitative interview of physicians	Objective: To study differences in individual physicians’ EHR use patterns and identify perceptions of uncertainty as an important variable in understanding EHR use	Qualitative assessment of physicians’ perceptions of uncertainty
Nevalainen, 2014^90^	Physician survey	Objective: To investigate medical students’ feelings about facing uncertainty in medical decision-making and the associations of tolerance of uncertainty using demographic factors, students’ fears of making mistakes and views of a GP’s work	Questionnaire about student’s views on how they felt about and tolerated uncertainty
Serbic, 2014^91^	Experimental study with patients (mixed factorial design)	Objective: To examine the relationship between diagnostic uncertainty and recall bias in 2 groups of chronic low back pain patients	Patients reported diagnostic uncertainty using the question, “I think there is something else happening with my back which the doctors have not found out about yet.” (yes/no)

**Table:** 

Take-Home Points – Diagnostic uncertainty lacks a clear definition, and there is no comprehensive framework for its measurement in medical practice. – Although different methods have been used to study diagnostic uncertainty in clinical practice, evidence is limited on which of these is the most useful or relevant. – We propose defining diagnostic uncertainty as a “*subjective perception of an inability to provide an accurate explanation of the patient’s health problem*.” – Methodological advancements in measuring diagnostic uncertainty can improve our understanding of diagnostic decision-making and inform interventions aimed at reducing diagnostic delays and overuse of health care resources.	

### Methods for Studying the Clinician’s Subjective Perception of Uncertainty

Multiple studies discussed uncertainty as an emotional response to complex situations (perceptions of and reactions to uncertainty) encountered by physicians, and attempted to measure it using personality tests and physician conversational analyses.^10^,^92^–^94^ These studies were not limited to diagnostic uncertainty, but also investigated uncertainty related to treatment, prognosis and other clinical decisions. Gerrity et al. developed a 23-item scale for physician reactions to uncertainty (PRU), which measures stress from decision-making in the midst of uncertainty.^40^,^66^ It comprises subscales for anxiety due to uncertainty, concern about bad outcomes, reluctance to disclose uncertainty to patients and reluctance to disclose mistakes to physician colleagues, and uses a six-point Likert scale ranging from “strongly disagree” to “strongly agree.”^41^ Similar scales were developed and validated in multiple studies for measuring tolerance for ambiguity (TFA) and physician risk attitude (PRA) in clinical practice.^72^,^81^,^83^,^90^,^95^ Cunningham used a five-item scale to measure emotional reactions and physician anxiety due to uncertainty (ADU Scale).^88^ Another qualitative study explored the relationship between physicians’ electronic health record (EHR) use patterns and their perceptions of uncertainty, and categorized physician users as uncertainty reductionists, uncertainty absorbers or hybrid users.^89^ Although not all of the above studies focused specifically on diagnosis, they provide an approach that could be used to understand the relationship between uncertainty and patient, clinician and organizational characteristics. Similar approaches have been used to evaluate relationships between the presence of uncertainty and resource use and physician work-related satisfaction.^9^,^21^,^76^

Some studies, however, have attempted to more explicitly measure *diagnostic* uncertainty versus all types of uncertainty (treatment, prognosis-related or other clinical decision-making). For example, Schneider et al., used the Dealing with Uncertainty Questionnaire (DUQ) consisting of a general practitioner (GP) diagnostic reasoning scale and GP action scale to study the diagnostic decision-making process and uncertainty.^42^,^82^ One study used a quantitative tool (survey) for physicians to self-report difficulties in making diagnoses.^85^ Three studies used diagnostic confidence as a measure for describing perceived uncertainty in diagnostic decision-making from the physician’s perspective.^74^,^86^,^96^ Studies also used qualitative approaches, including critical incident technique, grounded theory approach and semi-structured interviews, to ask clinicians about diagnostic decision-making in the midst of uncertainty.^49^,^73^,^77^,^80^,^97^

### Methods for Studying Uncertainty Within the Patient–Clinician Encounter

Multiple studies used evidence of unsolved diagnosis at a particular point in time (at discharge or end of visit) or proxy criteria to identify diagnostic uncertainty.^61^,^70^,^71^,^98^ For example, a criterion of three or more consultations at the end of a visit was used as an indirect measure of diagnostic uncertainty.^75^ Analyses of conversations, transcripts or video-recordings of patient–clinician encounters to identify words such as *probably*, *maybe* or *possibly* have been used to evaluate patterns suggestive of uncertainty.^38^,^72^,^92^ One study recorded expert clinicians while they were “thinking aloud” decisions in a case with uncertain diagnosis and analyzed transcripts for phrases that reflected uncertainty.^63^ Two studies measured clinician-documented uncertainty in retrospective chart reviews, using EHR data when either overly broad differential diagnoses or words such as *maybe* or *unclear* or question marks were reported in association with the diagnoses.^84^,^87^ A study by Lossos et al. examined administrative data using signs, symptoms and ill-defined conditions (SSIDs) codes based on the International Classification of Diseases, Ninth Revision, Clinical Modification (ICD-9 CM), and retrospective chart review, to measure diagnostic uncertainty in hospitalized patients.^64^ Standardized patients (actors trained to simulate real patients) depicting uncertain and complex situations have also been used.^67^ Although these studies focused specifically on uncertainty in diagnosis, information on the validity of methods was limited.

### Experimental Methods for Studying Uncertainty

Some studies used patient vignettes to measure diagnostic uncertainty from the patient’s perspective. These vignettes contained words and phrases that the physician might use to communicate uncertainty to the patient, and included direct expressions (e.g., “I cannot tell you,” “I do not know,” “I have difficulty answering this question”) or indirect expressions (probability statements—*maybe*, *probably*, *there is a good chance*, *might*, *may*, *should*—or conditional statements).^38^,^62^,^72^,^79^,^91^,^99^

### Approaches for Quantifying Diagnostic Uncertainty

Few attempts have been made to develop scales for quantifying diagnostic uncertainty; a recently published book on physician uncertainty in medicine discussed the spectrum of certainty, ranging from high confidence to pure speculation.^1^,^32^ Although multiple continuous or ordinal scales have been used, they have been applied differently. For example, one study asked clinicians to quantify their diagnostic uncertainty on a scale of 0 to 100 while treating a patient with dyspnea, where 0 and 100 represented complete certainty in ruling in or ruling out a diagnosis of heart failure, respectively. The study considered uncertainty to be present when physicians rated their certainty as between 21 and 79.^16^ Another study used a similar scale from 0 to 100 for each differential diagnoses considered, where 0 and 100 represented whether particular diagnoses were completely unlikely or likely, respectively.^69^ Ordinal scales used include visual analogs for the physician’s degree of uncertainty (e.g., 0, 25, 50, 75 and 100),^68^ or categories such as unknown, suspected, probable and certain; uncertain, moderately uncertain and certain; very little, some, moderate, substantial but not proven, and sufficient for proof; and low and high case uncertainty.^10^,^38^,^65^,^70^,^72^ Some studies have used similar approaches for quantifying the prevalence of uncertainty in different clinical settings (see supplement for details).

## DISCUSSION

Our review suggests that diagnostic uncertainty has yet to be clearly defined in the literature and lacks a robust measurement framework. This has impeded the design and development of validated instruments for measuring diagnostic uncertainty in medical practice. Based on our findings, we propose that diagnostic uncertainty be defined as a “*subjective perception of an inability to provide an accurate explanation of the patient’s health problem*.” The proposed definition aligns well with salient attributes discussed above and the recent National Academies for Science, Engineering and Medicine report on improving diagnosis,^21^ and can facilitate future efforts for identification and measurement of diagnostic uncertainty in medical practice.

### Operationalizing the Definition of Diagnostic Uncertainty

Several clinical and contextual factors need to be considered to operationalize our proposed definition for measurement. For instance, in the patient–clinician encounter, uncertainty experienced by a specific clinician regarding a patient’s health problem is dynamic and should be measured at a particular point in time (e.g., at the end of an encounter).^37^,^39^ Additionally, the clinician does not need to be absolutely certain about the diagnosis to initiate definitive treatment, but rather needs to reduce the level of diagnostic uncertainty below a certain threshold (Figure in Supplemental File) to narrow options to certain types of conditions.^32^,^50^,^100^–^102^ Thus, measurement should account for situations when the clinician is able to initiate definitive therapeutic care using a broader diagnosis, e.g., initiating symptomatic treatment for an upper respiratory infection of viral etiology in lieu of finding the more specific but clinically irrelevant information about the viral strain causing the infection. Further research will be needed to operationalize this proposed definition and to advance our understanding of diagnostic decision-making during uncertainty. This will expand our understanding of sources of diagnostic uncertainty and help inform robust conceptual frameworks to advance the “basic science” in this area.

### Methods for Measuring Diagnostic Uncertainty

Our review revealed several methods that have attempted to identify and measure uncertainty in medical practice. Validation studies have been largely limited to physician reactions to all types of uncertainty (diagnostic, treatment-related or prognostic), and few studies have focused exclusively on diagnostic uncertainty. One example involved a method for measuring physicians’ perceptions of diagnostic uncertainty (DUQ scale) which was validated in Germany and warrants additional evaluation in other clinical settings.^42^,^82^ Methods used to measure uncertainty more generally in practice might be tailored to measure diagnostic uncertainty specifically.^27^,^40^,^41^,^66^

We found that methods for measuring diagnostic uncertainty during the patient–clinician encounter are particularly underdeveloped. The increased availability of electronic clinical and administrative data related to the patient–clinician encounter and the rapid advances in health information technology capabilities (e.g., clinical algorithms, triggers, natural language processing) provide a new opportunity to develop and validate methods on a larger scale. Methods using experimental techniques also need further exploration. Some of the review findings could provide a foundation for developing and validating rigorous methods for identifying and measuring diagnostic uncertainty in medical practice. We need to develop both broad measurement approaches to identify certain signals or patterns in clinical decision-making across several diseases or conditions, as well as more specific approaches for disease-specific deep-dives.

### Implications for Clinical Practice

Efforts to identify and address diagnostic uncertainty are needed to improve clinical practice. First, because diagnostic uncertainty is ubiquitous, our findings suggest the need for clinicians to embrace it more fully, rather than the current norm of considering it as a negative concept.^5^ Second, more effective measurement and management of diagnostic uncertainty can potentially contribute to improvement of diagnostic decisionmaking and diagnostic safety. The recent report *Improving Diagnosis in Health Care* emphasized the importance of managing uncertainty during the process of diagnostic decision-making and the need for clinicians to acknowledge it in their daily work.^21^ Improved recognition of such uncertainty (such as documentation in the medical record) could help avoid diagnostic errors and aid clinicians in differentiating uncertain versus confirmed diagnosis during subsequent care. Additionally, communication that acknowledges uncertainty could facilitate closer follow-up of patients and ensure that they seek help if their condition is unchanged or worsens.

Third, measurement of diagnostic uncertainty can potentially improve our understanding of the efficiency of physician resource use and value-based health care delivery. Our review has several implications for controlling rising health care costs by improving physician decision-making related to ordering of diagnostic tests and use of specialty services.^103^ The “stubborn quest for diagnostic certainty” could be responsible for the dramatic variations in test use by physicians seeing similar patients.^11^,^40^,^50^,^104^–^106^ Identification and measurement of diagnostic uncertainty could potentially help reduce these variations. Current guidelines helping clinicians with test-ordering decisions (e.g., Choosing Wisely Campaign) have not yet reduced testing rates.^107^–^109^ Although guideline awareness could be a problem, variations in the magnitude of uncertainty perceived by the clinician could also be an explanatory factor.^107^–^109^ Nevertheless, much work remains to be done. While these studies imply an increase in health care costs and overuse of health care resources, none have measured the actual magnitude of increase. Qualitative methods were used in most studies to identify constructs and themes related to the presence of diagnostic uncertainty (e.g., “increase in resource use” and “increase in health care costs”).^9^–^15^ In addition, the effects of diagnostic uncertainty and delayed diagnosis in relation to morbidity, mortality and quality of life have been suggested, but not measured.^16^ Lack of a reliable definition of diagnostic uncertainty and valid measurement methods have made it difficult to calculate such estimates. Advances in measurement techniques would provide some numbers to reflect the magnitude of the problem.

Acknowledging diagnostic uncertainty while using these guidelines could also help clinicians deviate from guidelines in uncertain cases and justify the use (or non-use) of clinical resources (i.e., diagnostic tests, referrals, admissions or surgeries). For instance, the American College of Physicians recommends against imaging for nonspecific low-back pain in the absence of red flags.^110^ However, in the presence of diagnostic uncertainty (e.g., subjective fever and weight loss reported, but none measured objectively in clinic), it might be prudent to obtain imaging. Therefore, measuring uncertainty is essential for achieving an optimal “midpoint of the pendulum” between over-testing and under-diagnosis and in designing more clinically meaningful measures of value in care delivery.^111^

### Limitations

Our review has some limitations. We limited our key-word search strategy to two commonly used databases—Ovid Medline and PsycInfo—and only included articles in English, due to logistical limitations. We also limited the review to defining diagnostic uncertainty for the purpose of measurement in medical practice. As a result, we might have missed articles in which alternative means or methods were used to describe diagnostic uncertainty. However, we used a broad primary search strategy along with secondary search strategies to help capture key decision-making literature. We were also limited in our ability to assess the quality of each study. As with other reviews, the possibility of selective reporting and publication bias cannot be excluded.

## CONCLUSION

Diagnostic uncertainty, while prevalent, has not been comprehensively evaluated in current literature and medical practice. Although various methods have been used to study diagnostic uncertainty in clinical practice, evidence is limited as to which of these is the most useful or relevant, and no comprehensive measurement framework exists. On the basis of this review, we propose that diagnostic uncertainty be defined as “*subjective perception of an inability to provide an accurate explanation of the patient’s health problem*.” As next steps, we need to adopt a uniform definition of diagnostic uncertainty and work toward methodological advances in measuring diagnostic uncertainty in medical practice. The scientific foundation created in this review can inform future interventions aimed at improving the management of diagnostic uncertainty, thereby helping to reduce both under-diagnosis and overuse of health care resources.

## Electronic supplementary material

ESM 1 (DOCX 97 kb)
