# Supplementary material for: Defining and Measuring Diagnostic Uncertainty in Medicine: A Systematic Review
Source: J Gen Intern Med. 2017 Sep 21;33(1):103–15. doi: 10.1007/s11606-017-4164-1 (PMC5756158; doi:10.1007/s11606-017-4164-1)
Supplement: Supplementary file 1 — (DOCX 97 kb) [file 11606_2017_4164_MOESM1_ESM.docx]

**Supplemental Files**

**Supplemental Table 1: Search terms for diagnostic uncertainty**

| MeSH Headings | Free-Text terms |
| --- | --- |
| Diagnosis | Diagnos* Uncertain* (in 4 adjacent words) |
| Clinical Decision-Making | Diagnos* doubt* (in 4 adjacent words) |
| Delayed Diagnosis | Ambigu* diagnos*(in 4 adjacent words) |
| Diagnosis, Computer-Assisted | Diagnos* difficult* (in 4 adjacent words) |
| Diagnosis, Differential | Unsur* diagnos* (in 4 adjacent words) |
| Diagnostic Errors | unclear diagnos* (in 4 adjacent words) |
| Decision Making | vague diagnos* (in 4 adjacent words) |
| Uncertainty in Decision making | Question* diagnos* (in 4 adjacent words) |
| Mathematical Concepts, Probability, Uncertainty | ruleout diagnos* (in 4 adjacent words) |
| Epidemiological Methods, Uncertainty | Diagnos* confidence (in 4 adjacent words) |
|  | Diagnos* certain* (in 4 adjacent words) |

**Prevalence of diagnostic uncertainty**

Nine studies provided estimates of prevalence of diagnostic uncertainty in their respective study populations (1-9). Although studies on the prevalence of diagnostic uncertainty were limited in number, the prevalence is estimated to be between 1.5-25%. Because variations in case-mix and populations are common in clinical practice, these estimates might not be necessarily generalizable. This is not unexpected because of the subjective nature of diagnostic uncertainty (10-13) and the variable individual threshold to initiate action or treatment (also see Supplemental Figure) during the diagnostic process (11, 14-17). This threshold depends on clinician risk tolerance, experience and training (18). Future research focusing on understanding this diagnostic threshold and how to best quantify diagnostic uncertainty is warranted.

**Supplemental Table 2: Prevalence estimates of diagnostic uncertainty in medical practice**

| Citation | Setting and Country | Prevalence of Diagnostic Uncertainty |
| --- | --- | --- |
| Anstee, 1977 (1) | General hospital inpatient unit, England | 10% of all inpatient hospitalizations had uncertain diagnosis at discharge |
| Hartveit, 1979 (2) | Autopsy based study among cancer patients in Norway | Diagnosis was uncertain in 1 in 4 cases (25%) |
| Lossos, 1989 (3) | General hospital internal medicine unit, Israel | 1.8% of all admissions to an Internal Medicine department over a 9-year period had diagnostic uncertainty |
| Buntinx, 1991(4) | Primary care evaluation of patients presenting with chest pain or discomfort, Belgium | In 20% of patients, diagnosis was uncertain at initial evaluation. In 8%, diagnosis was uncertain at final diagnosis. |
| Haubek, 1991 (5) | Pathological diagnosis of renal mass lesion, Scandinavia | Diagnostic uncertainty was present in 19% of lesions. |
| Lave, 1997 (6) | General inpatient setting at an academic medical center, USA | 22.8% of patients had an uncertain initial admitting diagnosis. |
| Weijden, 2002 (7) | General surgery practice, Netherlands | 13% of the consultations had diagnostic uncertainty |
| Sarkar, 2012 (8) | Cross-sectional physician survey, USA | In at least 5% of cases seen by physicians, diagnosis was difficult and possibly uncertain |
| Whaley, 2013 (9) | Primary care evaluation of patients presenting with acute cough at an academic medical center, USA | Diagnostic uncertainty was present in 16% of patient visits. |

**Supplemental Figure: Hypothetical Scale of Diagnostic Uncertainty perceived by a physician diagnosing a specific patient at a particular point in time (11-13, 19)**


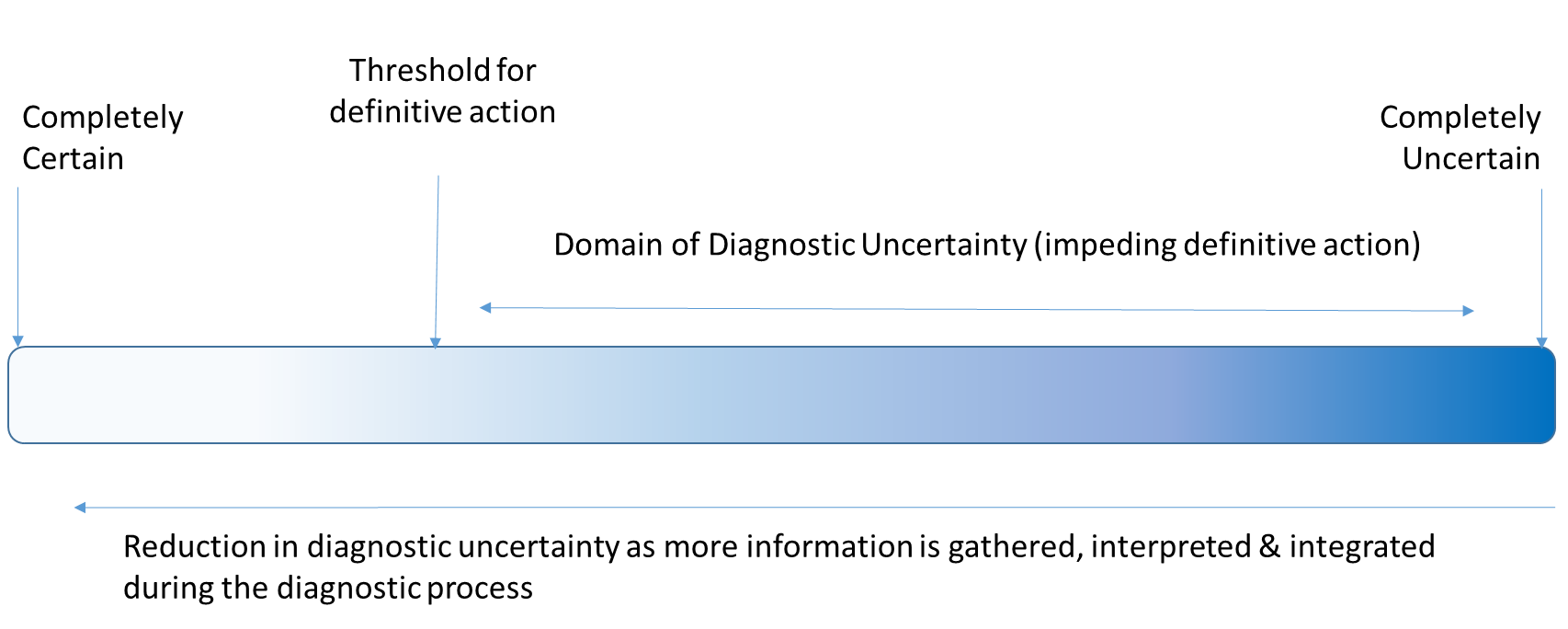


References

1. Anstee BH, Fleminger JJ. Diagnosis 'uncertain': A follow-up study. Br J Psychiatry. 1977 Dec;131:592-8. Available from:

2. Hartveit F. Autopsy findings in cases with a clinically uncertain cancer diagnosis. J Pathol. 1979 Nov;129(3):111-9. Available from:

3. Lossos I, Israeli A, Zajicek G, Berry EM. Diagnosis deferred--the clinical spectrum of diagnostic uncertainty. J Clin Epidemiol. 1989;42(7):649-57. Available from:

4. Buntinx F, Truyen J, Embrechts P, Moreel G, Peeters R. Chest pain: An evaluation of the initial diagnosis made by 25 flemish general practitioners. Fam Pract. 1991 Jun;8(2):121-4. Available from:

5. Haubek A, Lundorf E, Lauridsen KN. Diagnostic strategy in renal mass lesions. Scand J Urol Nephrol Suppl. 1991;137:35-9. Available from:

6. Lave JR, Bankowitz RA, Hughes-Cromwick P, Giuse NB. Diagnostic certainty and hospital resource use. Cost Qual Q J. 1997 26-32; quiz 46;3(3):22; Ju-23.

7. van der Weijden T, van Bokhoven MA, Dinant G, van Hasselt CM, Grol RPTM. Understanding laboratory testing in diagnostic uncertainty: A qualitative study in general practice. Br J Gen Pract. 2002 Dec;52(485):974-80.

8. Sarkar U, Bonacum D, Strull W, Spitzmueller C, Jin N, Lopez A, Giardina TD, Meyer AN, Singh H. Challenges of making a diagnosis in the outpatient setting: A multi-site survey of primary care physicians. BMJ Qual Saf. 2012 Aug;21(8):641-8.

9. Whaley LE, Businger AC, Dempsey PP, Linder JA. Visit complexity, diagnostic uncertainty, and antibiotic prescribing for acute cough in primary care: A retrospective study. BMC family practice. 2013;14(1):1.

10. Allman RM, Steinberg EP, Keruly JC, Dans PE. Physician tolerance for uncertainty. use of liver-spleen scans to detect metastases. JAMA. 1985 Jul 12;254(2):246-8.

11. Kassirer JP. Our stubborn quest for diagnostic certainty. N Engl J Med. 1989;320(22):1489-91.

12. Hatch, S. Snowball in a Blizzard: A Physician's Notes on Uncertainty in Medicine. Basic Books; 2016.

13. Reichenfeld HF. Certainty versus uncertainty in psychiatric diagnosis. Psychiatr J Univ Ott. 1990 Nov;15(4):189-93.

14. Heneghan C, Glasziou P, Thompson M, Rose P, Balla J, Lasserson D, Scott C, Perera R. Diagnostic strategies used in primary care. BMJ. 2009 Apr 20;338:b946.

15. Weinstein MC, Fineberg HV. Clinical decision analysis. . 1980

16. Pauker SG, Kassirer JP. Therapeutic decision making: A cost-benefit analysis. N Engl J Med. 1975;293(5):229-34.

17. Eisenberg JM, Hershey JC. Derived thresholds. determining the diagnostic probabilities at which clinicians initiate testing and treatment. Med Decis Making. 1983;3(2):155-68.

18. Zaat JO, van Eijk JT. General practitioners' uncertainty, risk preference, and use of laboratory tests. Med Care. 1992 Sep;30(9):846-54.

19. Djulbegovic B, Hozo I, Greenland S. Uncertainty in clinical medicine. Philosophy of Medicine. 2011;16:299.
